# Enhanced Thermoelectric Performance of n-Type Bi_2_Se_3_ Nanosheets through Sn Doping

**DOI:** 10.3390/nano11071827

**Published:** 2021-07-14

**Authors:** Mengyao Li, Yu Zhang, Ting Zhang, Yong Zuo, Ke Xiao, Jordi Arbiol, Jordi Llorca, Yu Liu, Andreu Cabot

**Affiliations:** 1 Catalonia Energy Research Institute—IREC, Sant Adrià de Besòs, 08930 Barcelona, Spain; limengyaorz@gmail.com (M.L.); tingchan99@gmail.com (Y.Z.); yongzuo16@gmail.com (Y.Z.); elvis.xiaoke@gmail.com (K.X.); 2 Catalan Institute of Nanoscience and Nanotechnology (ICN2), CSIC and BIST, Campus UAB, Bellaterra, 08193 Barcelona, Spain; ting.zhang@icn2.cat (T.Z.); arbiol@icrea.cat (J.A.); 3 Istituto Italiano di Tecnologia, Via Morego 30, 16163 Genova, Italy; 4 ICREA, Pg. Lluis Companys 23, 08010 Barcelona, Spain; 5 Institute of Energy Technologies, Department of Chemical Engineering and Barcelona Research Center in Multiscale Science and Engineering, Universitat Politècnica de Catalunya, EEBE, 08019 Barcelona, Spain; jordi.llorca@upc.edu; 6 Institute of Science and Technology Austria (IST Austria), Am Campus 1, 3400 Klosterneuburg, Austria

**Keywords:** thermoelectric, Bi_2_Se_3_, Sn doping

## Abstract

The cost-effective conversion of low-grade heat into electricity using thermoelectric devices requires developing alternative materials and material processing technologies able to reduce the currently high device manufacturing costs. In this direction, thermoelectric materials that do not rely on rare or toxic elements such as tellurium or lead need to be produced using high-throughput technologies not involving high temperatures and long processes. Bi_2_Se_3_ is an obvious possible Te-free alternative to Bi_2_Te_3_ for ambient temperature thermoelectric applications, but its performance is still low for practical applications, and additional efforts toward finding proper dopants are required. Here, we report a scalable method to produce Bi_2_Se_3_ nanosheets at low synthesis temperatures. We studied the influence of different dopants on the thermoelectric properties of this material. Among the elements tested, we demonstrated that Sn doping resulted in the best performance. Sn incorporation resulted in a significant improvement to the Bi_2_Se_3_ Seebeck coefficient and a reduction in the thermal conductivity in the direction of the hot-press axis, resulting in an overall 60% improvement in the thermoelectric figure of merit of Bi_2_Se_3_.

## 1. Introduction

Thermoelectric (TE) devices that directly and reversibly convert heat into electricity find unlimited applications [1,2,3,4,5,6], but their real implementation is hampered by their low cost-effectiveness. The efficiency of energy conversion of a TE device is in part determined by the transport properties of the TE material: Seebeck coefficient *S*, electrical conductivity *σ*, and thermal conductivity *κ*. These material properties are generally grouped into a dimensionless figure of merit, *ZT* = *S^2^σT/κ*, where *T* is the absolute temperature. Efficient TE materials are characterized by high power factors, *S^2^σ*, and low thermal conductivities, *κ*. However, the strong correlation between these parameters makes the optimization of the material performance extremely difficult. Several strategies have been developed to maximize *ZT*, including engineering the electronic band structure of the TE material through doping [7], the use of energy filtering interphases [8], and the reduction in lattice thermal conductivity through the introduction of abundant grain boundaries [9].

Commercial devices use large amounts of highly crystalline Bi_2_Te_3_-based alloys as the active TE material, which accounts for a significant part of the total cost of the device. To reduce the device cost, it is critical to develop low-cost materials not relying on scarce Te and low-cost processing strategies not based on high-temperature crystallization from high purity melts. Bi_2_Se_3_ presents the same structure and similar properties as Bi_2_Te_3_, and, thus, it is an obvious possible Te-free alternative to Bi_2_Te_3_. Numerous attempts to improve the *TE* figure of merit of this material have been reported, but with moderate success [10,11,12,13,14,15,16,17,18].

To maximize the TE performance of a material, one first critical point is to optimize its charge carrier concentration. Three main strategies can be used in this direction: tuning of the material stoichiometry, the introduction of atomic impurities, and/or the introduction of secondary phases able to inject the proper charge carriers to the host material [19,20,21,22,23,24,25,26,27,28]. Besides optimizing the charge carrier concentration, it is fundamental to minimize the thermal conductivity using multiscale phonon scattering centers. In this direction, the introduction of atomic impurities and abundant grain boundaries are highly effective. Besides, energy barriers at grain boundaries can also introduce energy- and charge sign-dependent charge carrier scattering, which could result in significant improvements to the Seebeck coefficient [29].

Here, we present a low temperature and high yield, solution-based strategy to prepare Bi_2_Se_3_ nanomaterials doped with different elements. The influence of different dopants on the TE properties of the material was tested. From the results obtained, we demonstrated Sn to have the highest potential to yield Bi_2_Se_3_-based materials with improved performance. Thus, we further analyzed the location and chemical state of this element within Bi_2_Se_3_ and its effect on the material transport properties.

## 2. Materials and Methods

### 2.1. Chemicals and Solvents

Polyvinylpyrrolidone (PVP, (C_6_H_9_NO)_n_, AMW ~55,000), indium(III) acetate (InC_6_H_9_O_6_, 99.99%), and bismuth(III) nitrate pentahydrate (Bi(NO_3_)_3_·5H_2_O, ≥99.99%) were purchased from Sigma-Aldrich. Sodium selenite (Na_2_SeO_3_, ≥98%), copper (II) nitrate trihydrate (Cu(NO_3_)_2_·3H_2_O, 99%), silver nitrate (AgNO_3_, ≥99.9%), ethylene glycol (EG, HOCH_2_CH_2_OH, 99%), lead (II) acetate trihydrate (PbC_4_H_6_O_4_·3H_2_O), tin(II) chloride anhydrous (SnCl_2_, 98%), and potassium hydroxide (KOH, ≥98%) were acquired from Fisher. Analytical grade ethanol and acetone were obtained from various sources.

### 2.2. Synthesis of Bi_2_Se_3_

Bi_2_Se_3_ particles were produced following the approach developed by Liu et al., with slight modifications [1]. Bi_2_Se_3_ (5 mmol), Bi(NO_3_)_3_·5H_2_O (10 mmol, 4.851 g), Na_2_SeO_3_ (15 mmol, 2.594 g), KOH (50 mmol, 2.806 g), and PVP (0.5 g) were first dissolved in a three-neck flask containing EG (200 mL) under an Ar atmosphere at an ambient temperature for 0.5 h. The solution was then heated to 180 °C and kept at this temperature for 3 h. Immediately after the reaction was completed, the solution was allowed to cool naturally to room temperature by removing the heating mantle. The solution was divided into several centrifuge tubes and acetone was added to collect the solid product by centrifugation. In the next step, ethanol was introduced to re-disperse the particles, and acetone was added to precipitate them again. This step was repeated twice. Next, purified Bi_2_Se_3_ particles were dried under a vacuum overnight at room temperature and kept in the glovebox for posterior use. Around 3 g of particles were obtained per batch.

### 2.3. Synthesis of Bi_2−__x_M_x_Se_3_

The procedure used to produce Bi_2−x_M_x_Se_3_ was the same used to produce Bi_2_Se_3_, but the proper amount of bismuth nitrate was replaced with the corresponding metal precursor, as listed in the chemical section above.

### 2.4. Nanomaterial Consolidation

Dried Bi_2_Se_3_ particles were first annealed for 1 h at 350 °C under an Ar flow in a tubular furnace. Then, the annealed material was introduced into a graphite die and consolidated into cylinders (Ø 10 mm × 10 mm) at 480 °C and 50 MPa of pressure for 4 min with a custom-made hot press. The relative densities of the compacted pellets were measured by Archimedes’ method. To measure transport properties in the two relevant directions, cylindrical pellets were cut into ca. 8 × 6 × 1 mm rectangular bars along the pressure axis, and 1 mm thick disks along the perpendicular to the pressure axis.

### 2.5. Structural and Chemical Characterization

The field emission scanning electron microscope (SEM) was used to determine the nanoparticle morphology on an Auriga Zeiss. An Oxford energy dispersive X-ray spectrometer (EDX) was used to measure the material composition at 20.0 kV. X-ray diffraction (XRD) was performed on a Bruker AXS D8 Advance diffractometer. The crystal structure of the samples was analyzed under the 200 keV Tecnai F20 field emission microscope equipped with a Gatan quantum image filter. X-ray photoelectron spectroscopy (XPS) was performed on a Specs system with the material inside the chamber at a pressure below 10^−7^ Pa. Data processing was carried out using the CasaXPS program.

### 2.6. Performance Characterization of Bulk Nanomaterial

Seebeck coefficients and resistivities were measured by the static direct current method and by the standard four-probe method, respectively, in an LSR-3 Linseis system under helium. All samples were tested for at least three heating and cooling cycles. Considering the system accuracy and measurement accuracy, the measurement error of the conductivity and Seebeck coefficient was estimated to be about 4%. Thermal diffusivity (λ), constant pressure heat capacity (C_p_), and density of the material (ρ) were used to obtain the thermal conductivities (κ_total_), where κ_total_ = λC_p_ρ. The thermal diffusivities of the samples were measured by a Xenon Flash Apparatus XFA600, which has an estimated error of ca. 5%. We used a constant as the heat capacity (C_p_), which was estimated from empirical formulas by the Dulong–Petit limit (3R law). Under a magnetic field of 0.6 T, the Hall charge carrier concentrations and mobilities at room temperature were measured by the Van der Pauw method. The figures in this article do not have error bars in order to avoid cluttering the plots.

## 3. Results and Discussion

**Figure 1:**
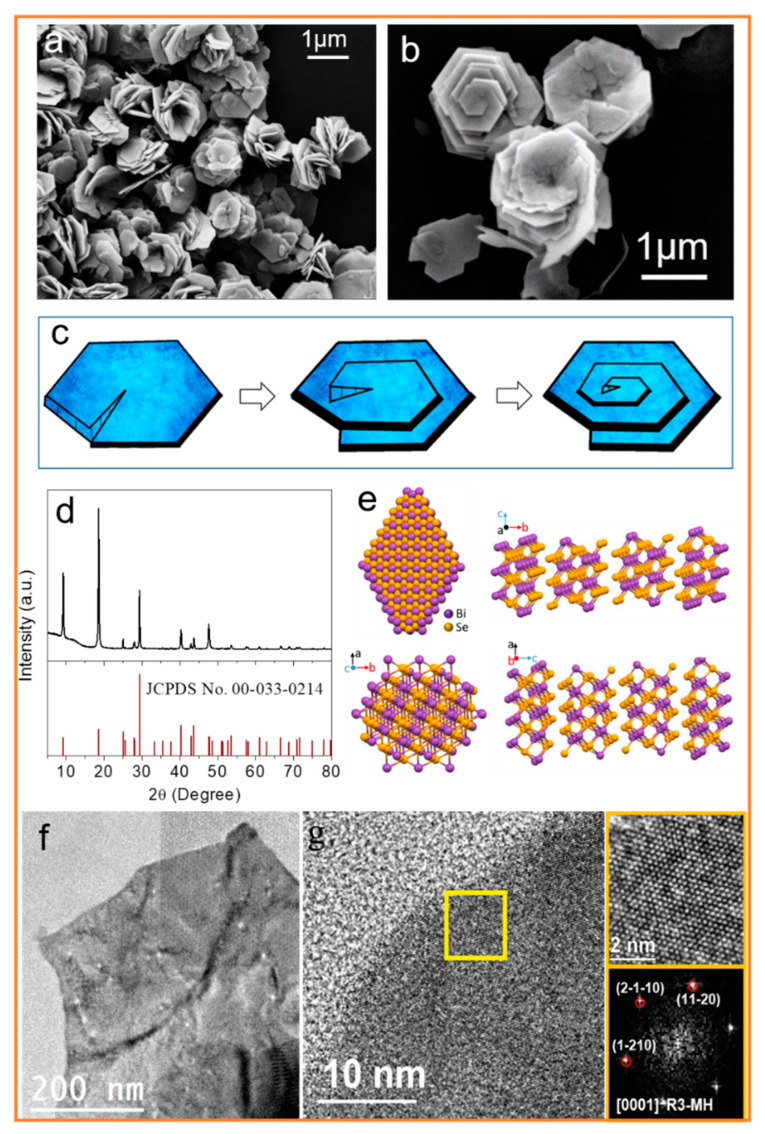
(**a**,**b**) SEM micrographs of the Bi_2_Se_3_ particles. (**c**) Scheme of the screw dislocation growth mechanism. (**d**) The XRD pattern of the Bi_2_Se_3_ particles. (**e**) The layered rhombohedral crystal structure of Bi_2_Se_3_. (**f**) A low-resolution TEM image. (**g**) An HRTEM micrograph of the Bi_2_Se_3_ particles and its corresponding power spectrum. From the crystallographic domain, the Bi_2_Se_3_ lattice fringe distances were measured to be 0.205 nm, 0.205 nm, and 0.204 nm, at 60.96° and 120.86°, respectively, which could be illuminated as the rhombohedral Bi_2_Se_3_ phase, visualized along the [0001] zone axis.

Following the above synthesis method, Bi_2_Se_3_ nanosheets grouped into flower-like particles were produced, as shown by the SEM micrographs (Figure 1a,b). Bi_2_Se_3_ shows a screw dislocation growth mechanism (Figure 1c). This layer-by-layer growth mechanism is usually due to the low supersaturation in the synthesis conditions, which inhibits dendritic growth. Axial screw dislocation is the self-sustaining spiral growth of nanosheets around the axis [15,23].

The XRD analysis showed that the crystal structure of the obtained nanosheets matched the rhombohedral Bi_2_Se_3_ phase (Figure 1d, JCPDS No. 00-033-0214). Figure 1e shows the layered rhombohedral crystal structure of Bi_2_Se_3_, which consists of five covalently bonded atomic planes of Se-Bi-Se-Bi-Se. Quintuple layers are weakly bonded by van der Waals. The HRTEM characterization showed that the material has good crystallinity and has a crystal phase consistent with the Bi_2_Se_3_ rhombohedral phase (space group = R3-MH) with a = b = 4.1340 Å and c = 28.6300 Å (Figure 1f,g).

**Figure 2:**
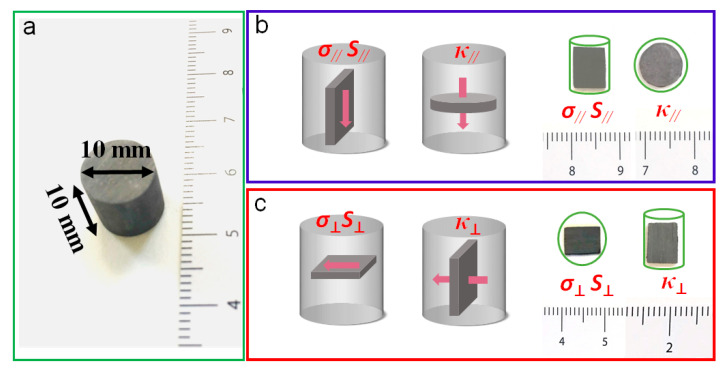
(**a**) Photograph of the consolidated Bi_2_Se_3_ cylinder. (**b**,**c**) Scheme and images of the samples used to measure the material transport properties in the directions parallel (**b**) and vertical (**c**) to the pressure axis.

Nanosheets were annealed at 350 °C, and the annealed powder was hot-pressed into cylindrical pellets with a diameter of 10 mm and a height of 10 mm in a glovebox filled with argon. Samples were hot-pressed for four minutes at 480 °C and 50 MPa, and then naturally cooled to an ambient temperature. The relative density of the cylinders produced by this process was about 93% of the theoretical value. From the consolidated cylinder, a rectangular bar of 8 × 6 × 1 mm was cut longitudinally, and a 1 mm thick pellet was cut transversely (Figure 2). These samples were used to measure the TE properties of materials parallel to the pressure axis (//) and perpendicular to the pressure axis (⊥).

**Figure 3:**
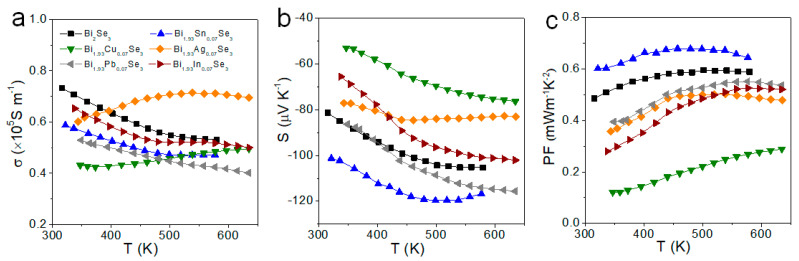
TE properties of Bi_2−x_M_x_Se_3_ (M = Sn, Cu, Ag, Pb, In) samples measured perpendicular to the press direction: (**a**) electrical conductivity, *σ*; (**b**) Seebeck coefficient, *S*; (**c**) power factor, *PF*.

Figure 3 displays the electrical conductivity (*σ*), Seebeck coefficient (*S*), and power factor (*PF* = *S*^2^*σ*) of the Bi_2−x_M_x_Se_3_ (M = Sn, Cu, Ag, Pb, In) pellets perpendicular to the press direction. The electrical conductivity of the undoped material decreased with temperature, which pointed to a degenerated semiconductor behavior. Compared to the undoped material, the electrical conductivity of all the doped samples was slightly lower, except for the Ag-doped samples. On the other hand, the samples doped with Sn and to a minor extent, Pb displayed an increase in the absolute value of the Seebeck coefficient. Overall, the highest power factors were obtained with the Sn doping. Thus, we decided to further study the effect of this element.

**Figure 4:**
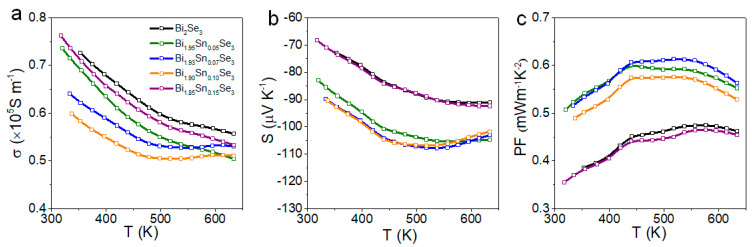
Thermoelectric properties of Bi_2−x_Sn_x_Se_3_ perpendicular to the press direction: (**a**) electrical conductivity, *σ*; (**b**) Seebeck coefficient, *S*; (**c**) power factor, *PF*.

Figure 4 displays the electrical conductivity, Seebeck coefficient, and power factor of Bi_2-x_Sn_x_Se_3_ materials containing different amounts of Sn. We observed that the electrical conductivity decreased and the absolute value of the Seebeck coefficient increased with the amount of Sn up to a certain Sn concentration. The highest power factors were finally obtained for Bi_1.93_Sn_0.07_Se_3_. The decrease in the electrical conductivity and increase in the absolute value of the Seebeck coefficient denoted a reduction in the charge carrier concentration, which points to the presence of Sn^2+^ ions instead of Sn^4+^ at Bi^3+^ sites.

**Figure 5:**
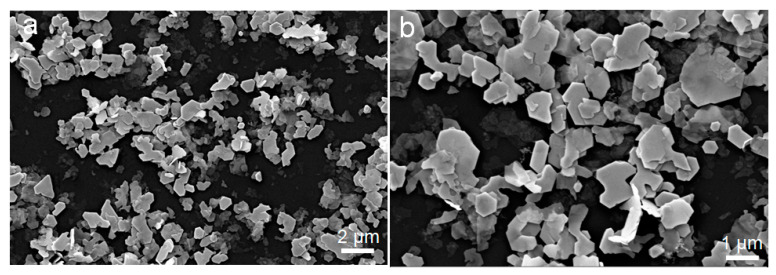
SEM micrographs of Bi_1.93_Sn_0.07_Se_3_ nanosheets with different magnifications; (**a**) magnification 5.00 K ×, (**b**) magnification 10.00 K ×.

**Figure 6:**
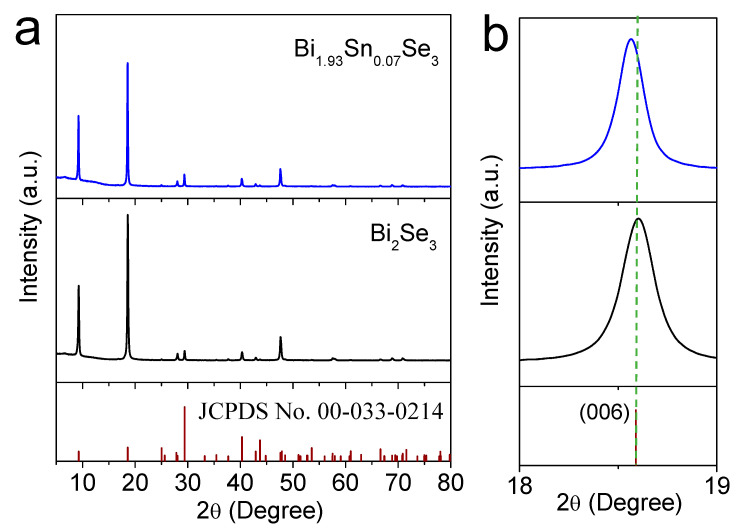
(**a**) XRD patterns of Bi_2_Se_3_ and Bi_1.93_Sn_0.07_Se_3_; (**b**) expansion diagram of the area corresponding to the diffraction peak of Bi_2_Se_3_ (006).

Figure 5 shows representative SEM micrographs of the Bi_1.93_Sn_0.07_Se_3_ particles, which had similar sizes but did not display the flower-like morphology observed from Bi_2_Se_3_. As shown in Figure 6, the XRD characterization confirmed that the Bi_1.93_Sn_0.07_Se_3_ particles maintained their rhombohedral structure. With the introduction of Sn, the XRD peaks shifted to lower 2θ angles, suggesting the incorporation of Sn within the Bi_2_Se_3_ lattice [5].

**Figure 7:**
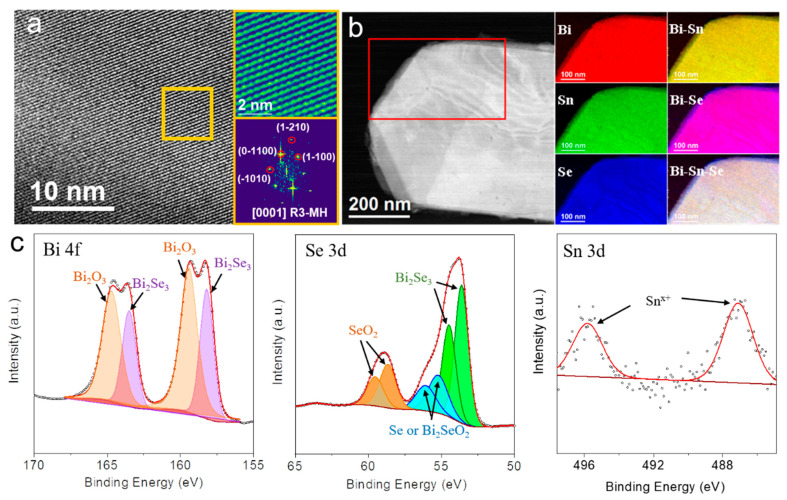
(**a**) An HRTEM micrograph of a Bi_1.93_Sn_0.07_Se_3_ nanosheet, detailed information of the orange squared area and its corresponding power spectrum. Bi_2_Se_3_ lattice fringe distances were measured to be 0.370 nm, 0.349 nm, 0.202 nm, and 0.351 nm, at 62.89°, 92.61° and 122.99°, respectively, which could be illuminated as the rhombohedral Bi_2_Se_3_ phase, along its [0001] zone axis. (**b**) EELS chemical composition maps obtained from the red squared area of the STEM micrograph. Individual Bi N_2,3_-edges at 679 eV (red), Sn M_4,5_-edges at 485 eV (green), Se M_2,3_-edges at 162 eV (blue) and composites of Bi-Sn, Bi-Se as well as Bi-Sn-Se. (**c**) Bi 4f, Se 3d and Sn 3d high-resolution XPS spectra obtained from Bi_1.93_Sn_0.07_Se_3_ nanosheets.

The HRTEM characterization confirmed the rhombohedral phase of the Bi_1.93_Sn_0.07_Se_3_ particles (Figure 7a). EELS chemical composition maps obtained from the red squared region in the HAADF STEM micrograph shown in Figure 7b displayed a homogeneous distribution of Sn, Bi and Se within the Bi_1.93_Sn_0.07_Se_3_ nanosheet.

Bi 4f, Se 3d and Sn 3d high-resolution XPS spectra of Bi_1.93_Sn_0.07_Se_3_ particles are displayed in Figure 7c. The high-resolution Bi 4f spectrum was fitted with two doublets, associated with Bi^3+^ within a Bi_2_Se_3_ chemical environment (Bi 4f_7/2_ at 158.2 eV) and Bi within a more electronegative environment, as it could be Bi_2_O_3_ or Bi_2_SeO_2_ (Bi 4f_7/2_ at 159.4 eV) [30]. This oxidation of the particles surface is related to their transport and handling in the air [4]. The high-resolution Se 3d XPS spectrum was fitted with three doublets, corresponding to Se within the Bi_2_Se_3_ (Se 3d_5/2_ at 53.6 eV), selenium in an elemental or Bi_2_SeO_2_ environment (Se 3d_5/2_ at 55.2 eV), probably arising from the partial oxidation of the material surface, and Se in a SeO_2_ chemical environment (Se 3d_5/2_ at 58.6 eV) [30,31]. Finally, the high-resolution Sn 3d XPS spectrum was difficult to fit owing to the small amount of this element, but the broadness of the peaks pointed to the presence of at least two Sn chemical states that should be tentatively assigned to Sn^x+^ within a SnSe and an oxidized environment [31]. According to the electrical properties measured at the introduction of Sn, we tentatively assigned the Sn^x+^ component within SnSe to Sn^2+^.

**Figure 8:**
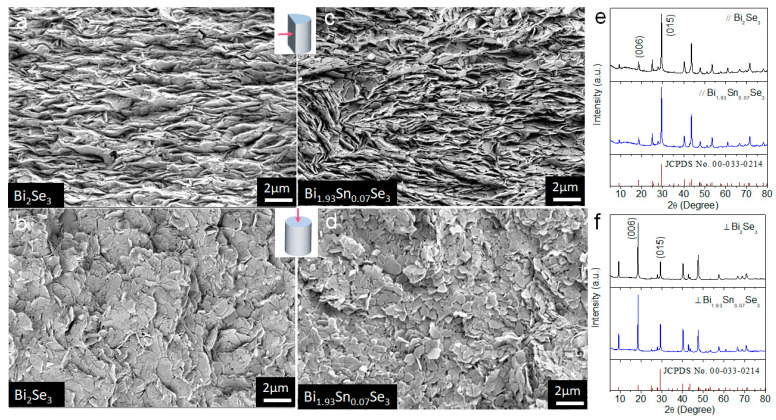
(**a**,**b**) Cross-section and top-view SEM micrograph of Bi_2_Se_3_. (**c**,**d**) Cross-section and top-view SEM micrograph of Bi_1.93_Sn_0.07_Se_3_. (**e**,**f**) XRD patterns of Bi_2_Se_3_ and Bi_1.93_Sn_0.07_Se_3_ measured in two perpendicular directions, along (//) and perpendicular (⊥) to the pressure axis.

Top-view and cross-section SEM micrographs of Bi_2_Se_3_ and Bi_1.93_Sn_0.07_Se_3_ (Figure 8) showed the final pellets presenting a laminar microstructure with an evident preferential orientation of the material layers. Bi_2_Se_3_ displayed larger and thinner layers than Bi_1.93_Sn_0.07_Se_3_. When comparing the XRD patterns of the sample held in two normal directions, parallel and perpendicular to the pressure axis (Figure 8e,f), we observed that the relative XRD peak intensity clearly differed. This result confirmed the preferential crystallographic orientation of the hot-pressed materials, with the [001] crystallographic direction oriented parallel to the pressure axis.

**Figure 9:**
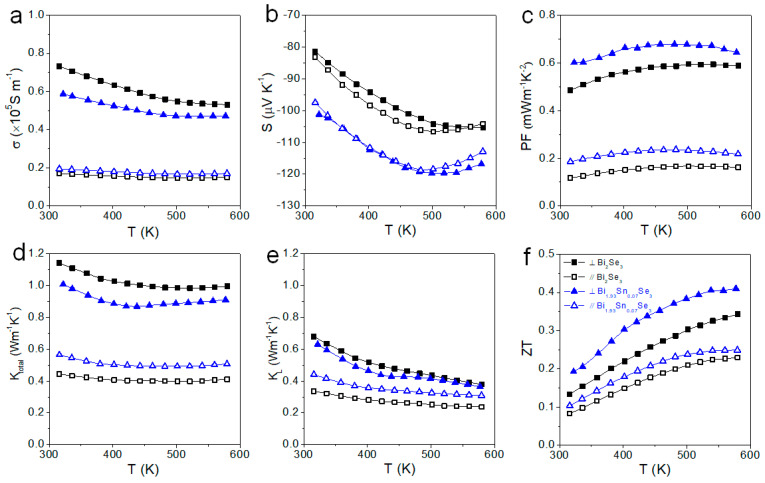
TE properties of Bi_2_Se_3_ and Bi_1.93_Sn_0.07_Se_3_ parallel (//) and vertical (⊥) to the pressure axis: (**a**) electrical conductivity, *σ*; (**b**) Seebeck coefficient, *S*; (**c**) power factor, *PF*; (**d**) total thermal conductivity, *κ*; (**e**) lattice thermal conductivity, *κ_L_*; and (**f**) the TE figure of merit, *ZT*.

Figure 9 displays the TE properties of Bi_2_Se_3_ and Bi_1.93_Sn_0.07_Se_3_ measured in the two directions. We observed *σ*_⊥_ > *σ*_//_, as expected from the higher charge carrier mobilities in the *ab* crystal plane compared with the *c* direction, and the extended size of the crystal domains in the direction normal to the pressure direction within the layered pellets. On the other hand, similar Seebeck coefficients were obtained in both directions, pointing out that this parameter has little relationship with grain boundary scattering in this material.

When comparing the TE properties of Bi_2_Se_3_ and Bi_1.93_Sn_0.07_Se_3_, we observed *σ*_⊥_ to slightly increase and *σ*_//_ to slightly decrease with the introduction of Sn, which could be in part related to the thinner and larger material layers observed within the layered Bi_2_Se_3_ pellets compared with Bi_1.93_Sn_0.07_Se_3_. On the other hand, with the introduction of Sn, *S* increased in both directions, which resulted in a higher *PF* in both directions for the Bi_1.93_Sn_0.07_Se_3_ sample.

Table 1 displays the Hall charge carrier concentration and mobility of Bi_2_Se_3_ and Bi_1.93_Se_0.07_Se_3_ pellets at room temperature. As expected from the electrical conductivity and Seebeck coefficient measurements, we observed the Sn introduction to result in a decrease in the charge carrier concentration, from *n_H_* = 1.8 × 10^19^ cm^−3^ for Bi_2_Se_3_ to *n_H_* = 1.3 × 10^19^ cm^−3^ for Bi_1.93_Se_0.07_Se_3_. This result is consistent with the presence of Sn^2+^ replacing Bi^3+^ ions, thus trapping a free electron. We believe this optimization of the charge carrier concentration to be at the origin of the higher *PF* obtained with the introduction of Sn. We further calculated the effective mass of the Bi_2_Se_3_ and Bi_1.93_Sn_0.07_Se_3_ materials using a single parabolic band (SPB) model. For detailed calculations, the carrier transport property analysis included [32]:

The Seebeck coefficient,

(1) $$S\left(\eta \right)=\frac{\kappa_{B}}{e}\left[\frac{\left(r+5/2\right)\cdot F_{r+3/2}\left(\eta \right)}{\left(r+3/2\right)\cdot F_{r+1/2}\left(\eta \right)}-\eta \right]\;$$

The Hall carrier concentration,

(2) $$n_{H}=\frac{1}{e\cdot R_{H}}=\frac{{\left(2m^{*}\cdot \kappa_{B}T\right)}^{3/2}}{3\pi^{2}\hbar^{3}}\cdot \frac{{\left(r+3/2\right)}^{2}\cdot F_{r+1/2}^{2}\left(\eta \right)}{\left(2r+3/2\right)\cdot F_{2r+1/2}\left(\eta \right)}\;$$

The Hall mobility,

(3) $$\mu_{H}=\left[\frac{e\pi \hbar^{4}}{\sqrt{2}{\left(\kappa_{B}T\right)}^{3/2}}\frac{C_{l}}{E_{def}{}^{2}{\left(m^{*}\right)}^{5/2}}\right]\frac{\left(2r+3/2\right)\cdot F_{2r+1/2}\left(\eta \right)}{{\left(r+3/2\right)}^{2}\cdot F_{r+1/2}\left(\eta \right)}\;$$

where $F_{x}\left(\eta \right)=\int_{0}^{\infty }\frac{\varepsilon^{x}}{1+e^{\left(\varepsilon -\eta \right)}}d\varepsilon \;$ is the Fermi integral.

In the above equations, *S*, *μ_H_*, *η*, *κ_B_*, *e*, *r*, *R_H_*, *ℏ*, *C_l_*, *E_def_*, and m* are the Seebeck coefficient, the carrier mobility, the reduced Fermi level, the Boltzmann constant, the electron charge, the carrier scattering factor (*r* = −1/2 for acoustic phonon scattering), the Hall coefficient, the reduced plank constant, the elastic constant for longitudinal vibrations, the deformation potential coefficient, and the density of state effective mass, respectively. Using the experimental *S*, the reduced Fermi level *η* can be obtained by Equation (1). Substituting the estimated *η* and the measured “*n_H_*” and “μ*_H_*” into Equations (2) and (3), the effective mass “m*” can be calculated. The results show that m* increased slightly with the Sn doping (Table 1).

Consistent with the layered structure of the pellets and their crystallographic texture, the thermal conductivity of the materials measured in the direction normal to the pressure axis was much higher than in the pressure axis, *κ*_⊥_ > *κ*_//_. The introduction of Sn resulted in lower *κ*_⊥_ but higher *κ*_//_ values, which is related to the different microstructure of the layers, with thicker and smaller Bi_1.93_Se_0.07_Se_3_ plates compared to those within the Bi_2_Se_3_ pellet.

Overall, the Bi_1.93_Sn_0.07_Se_3_ samples measured in the pellet plane displayed the highest PF and ZT values, 0.65 m Wm^−1^ K^−2^ and 0.41, respectively. These values are among the highest published for Bi_2_Se_3_-based materials, as displayed in Table 2.

## 4. Conclusions

We report here a large-scale method to synthesize n-type Bi_2_Se_3_ nanosheets with different dopants. Samples doped with Sn provided the highest TE performances. The composition was optimized at Bi_1.93_Se_0.07_Se_3_. Upon Sn doping, we observed an increase in the Seebeck coefficient and the power factor compared to pure Bi_2_Se_3_. As a result, a power factor up to 0.65 m Wm^−1^K^−2^ and a ZT of 0.41 were obtained for the Bi_1.93_Sn_0.07_Se_3_ pellet measured in the direction perpendicular to the pressure axis, which represents a 60% increase over pure Bi_2_Se_3_.

## Figures and Tables

**Table 1:** Room temperature transport properties and m* of the Bi_2_Se_3_ and Bi_1.93_Se_0.07_Se_3_ pellets.

Materials	*n_H_* [10^19^ cm^−3^]	μ*_H_* [cm^2^ V^−1^ S^−1^]	m*/*m*_0_
Bi_2_Se_3_	1.77	178.6	0.27
Bi_1.93_Sn_0.07_Se_3_	1.34	239.3	0.29

**Table 2:** A comprehensive summary of the thermoelectric performance of the previously reported Bi_2_Se_3_.

	ZT at Room Temperature				ZT_max_			
Materials	*σ* (10^4^S/m)	*S* (μV/K)	*κ* (W/mK)	ZT	*σ* (10^4^ S/m)	*S* (μV/K)	*κ* (W/mK)	ZT
K_2.5_Bi_8.5_Se_14_ [33]	0.4	−100	0.6	0.05	1.8	−160	0.38	1 (873 K)
Bi_0.7_Sb_0.3_Se [34]	4.5	−138	0.5	0.45	4.2	−160	0.7	0.8 (425 k)
Bi_5.6_Sb_2.4_Se_7_ [35]					4.8	−168.5	0.73	0.55 (300 K)
Bi_2_Se_3_ [28]	2	−120	0.5	0.17	1.95	−155	0.4	0.48 (427 K)
Bi_2_Se_3_ [21]	1.78	−90	0.42	0.1	3	−120.7	0.49	0.35 (400 K)
Bi_2_Se_3_ HA_0.11_DMSO_0.06_ [36]	14.8	−80	1.52	0.187				
Bi_2_Se_3_ [12]	2.5	−60	0.55	0.05	2	−100	0.6	0.18 (480 K)
Cu_0.1_Bi_2_Se_3_ [37]					1.46	−84	0.32	0.1 (290 K)
Bi_2_Se_3_ [20]	0.212	−115	0.75	0.011	0.6755	−150	0.83	0.096 (523 K)
**This work**	**5.87**	**−101**	**1**	**0.195**	**4.72**	**−116**	**0.91**	**0.41** (577 K)

## Data Availability

Data are contained within the article.
